# Evaluation of HPV infection and presence of licensed HPV vaccine genotypes among genital warts in Foshan, China

**DOI:** 10.3389/fmicb.2024.1376141

**Published:** 2024-04-17

**Authors:** Zeqi Huang, Shaonuan Yao, Lin Zou, Weixian Xie, Dongde Xie, Weiwei Li, Deyou Tan, Jiangang Shuai

**Affiliations:** ^1^Department of Clinical Laboratory, The Second People’s Hospital of Foshan, Foshan, China; ^2^Department of Medical Records, The Second People’s Hospital of Foshan, Foshan, China; ^3^Department of Dermatology, The Second People’s Hospital of Foshan, Foshan, China; ^4^Department of Gynaecology, The Second People’s Hospital of Foshan, Foshan, China

**Keywords:** human papillomavirus, infection, HPV vaccine, genotype, genital warts

## Abstract

**Objective:**

This study aimed to evaluate the prevalence of human papillomavirus (HPV) infection and presence of licensed HPV vaccine genotypes among patients with genital warts in Foshan, China from 2015 to 2022, to provide useful references for the detection, prevention and control of genital warts in Foshan.

**Methods:**

The present study retrospectively analyzed the HPV detection rates in patients with genital warts. A total of 1,625 patients were seen at the Second People’s Hospital of Foshan, Guangdong Province, China, from 2015 to 2022. Samples were collected from various lesions and genotyped for 21 genotypes of HPV by infusion hybridization. The classification principle of HPV genotypes in this study: (1) Based on the relationship between HPV and carcinogenicity; (2) Based on the number of HPV genotypes infected; (3) Based on the HPV genotypes of licensed HPV vaccines.

**Results:**

The detection rate of any HPV in patients with genital warts was 80.37% (1,306/1,625). The detection rates of HPV for low-risk infection, co-infection and high-risk infection were 49.48% (804/1,625), 24.92% (405/1,625) and 5.97% (97/1,625), respectively. Single infection was the predominant type (51.94%, 844/1625). HPV-6 and HPV-11 were the predominant types of single infection; HPV-6 and HPV-52 were the predominant types of paired combinations of multiple infection. 82.22% (1,336/1,625) of the cases had an age distribution of ≤ 24, 25–34, and 35–44. The distribution of some HPV genotypes had age specificity, annual specificity and gender specificity. The genotype detection rates of 2v, 4v and 9v showed a decreasing trend with ages (all *P* < 0.05). The genotype detection rates of 4v and 9v showed a decreasing trend over the 8-year period (both *P* < 0.05). The genotype detection rates of 4v and 9v in the male group were higher than those in the female group (both *P* < 0.05). The genotype detection rate of 9v was significantly higher than that of 2v and 4v in the female group (both *P* < 0.05).

**Conclusion:**

Our study demonstrated that low-risk infection and single infection were the main types of HPV infection in patients with genital warts, mainly among young patients. Our study provides epidemiological data for the detection, prevention and control of genital warts in China.

## 1 Introduction

Human papillomavirus (HPV) is a round, non-enveloped, double-stranded DNA virus belonging to the family papillomaviridae, whose genome consists of approximately 8,000 bases (Owczarek et al., 2021). It is epithelialized. Based on their oncogenic potential, they are mainly classified into 2 major groups: high-risk HPV (HR-HPV) and low-risk HPV (LR-HPV) (Stanley, 2007). HR-HPV such as HPV-16 and HPV-18, are the main causes of cervical precancerous lesions and cervical cancer (Crow, 2012). LR-HPV such as HPV-6 and HPV-11 are responsible for over 90 percent of the genital warts (Steben and Garland, 2014). Genital warts also known as condyloma acuminatum (CA) or venereal warts, is a sexually transmitted disease caused by human papillomavirus, which mainly occurs in the genital area of the skin mucosa, and is harmful to human health. LR-HPV can cause benign genital warts, such as condyloma acuminatum, and the probability of causing malignant transformation is relatively low. HR-HPV causes hyperproliferation of keratinocytes in lesions, which can lead to cancer in severe cases (Medda et al., 2021). Recent studies highlight the complex interplay between high-risk and low-risk HPV types, suggesting that this interaction may significantly elevate the risk of genital warts development. This nuanced understanding of HPV type interactions underscores the importance of comprehensive HPV typing in clinical and epidemiological investigations of HPV-related diseases (Hasanzadeh et al., 2019; Malagutti et al., 2020).

The attention to HPV vaccine is showing an increasing trend year by year (Bhagavathula and Massey, 2022). Genital warts can be prevented by HPV vaccination, and 2 licensed vaccines related to low-risk types of HPV, the quadrivalent HPV vaccine (4vHPV, Gardasil-4, HPV-6/11/16/18) and the nine-valent HPV vaccine (9vHPV, Gardasil-9, HPV-6/11/16/18/31/33/45/52/58), can effectively prevent HPV-6 and HPV-11 infections (Geretti et al., 2016). There are five types of HPV preventive vaccines that have completed relevant clinical trials in China and have been proven to have good tolerance and immunogenicity: bivalent HPV vaccine (Cervarix, approved in 2016, WHO), quadrivalent HPV vaccine (Gardasil, approved in 2017, WHO), nine-valent HPV vaccine (Gardasil-9, approved in 2018, WHO), homemade *Escherichia coli* produced HPV bivalent vaccine (Cecolin, approved in 2019, China), and *Pichia pastoris* produced HPV bivalent vaccine (Walrinvax, approved in 2022, China) (Li et al., 2023). A summary analysis of 1.7 million ordinary women in China showed that the overall prevalence of HPV infection in the country was 15.54% (95% CI: 13.83–17.24%). The top 5 common HPV types detected in the general population were HPV-16 (3.52%, 95% CI: 3.18–3.86%), HPV-52 (2.20%, 95% CI: 1.93–2.46%), HPV-58 (2.10%, 95% CI: 1.88–2.32%), HPV-18 (1.20%, 95% CI: 1.05–1.35%), and HPV-33 (1.02%, 95% CI: 0.89–1.14%) (Zhu B. et al., 2019). Countries with high HPV vaccination coverage have reported a decrease in the burden of genital warts in young women (Smith et al., 2015). However, only 11 countries have included men (World Health Organization [WHO], 2017). According to recent studies, 1/3 of men worldwide are infected with at least one type of genital HPV. Sexually active men, regardless of age, are important hosts for genital HPV infections (Bruni et al., 2023). Prevention and treatment of human papillomavirus in men benefits both men and women (Zou et al., 2022).

At present, research on HPV infection mainly focuses on female, and HPV vaccination mainly prevents cervical cancer in female. The development of licensed vaccines is mainly based on epidemiological data from western countries, and they cannot prevent all prevalent HPV genotypes. Therefore, this study evaluated the epidemiological data of human papillomavirus infection and presence of licensed HPV vaccine genotypes among genital warts patients (including males) in Foshan, providing useful references for the detection, prevention, and control of related genital warts in Foshan.

## 2 Materials and methods

### 2.1 Study population

This study was conducted from January 2015 to December 2022 in the Second People’s Hospital of Foshan. The study enrolled a total of 1,625 patients, comprising 48.06% males and 51.94% females. The age of the participants ranged from 15 to 87 years, with a median age of 31 years. This demographic information is crucial for contextualizing our findings within the broader scope of HPV prevalence and vaccine coverage discussions. The study was conducted in accordance with the declaration of Helsinki and approved by the Ethics Committee of the Second People’s Hospital of Foshan. The inclusion criteria were as follows: (1) All cases met the clinical diagnostic criteria in the Chinese Guidelines for the Diagnosis and Treatment of genital warts (2014); (2) All cases had an epidemiologic history with typical clinical manifestations; (3) All cases had resided in Foshan, Guangdong Province; (4) All cases had signed an informed consent form and voluntarily participated in this study. The exclusion criteria were as follows: (1) Cases with abnormal sample collection or undetected internal standards; (2) Cases with incomplete medical information, such as name, age, gender, visit time, and clinical diagnosis; (3) Cases with genital or vulvar cancer.

### 2.2 Sample collection

Patients were clinically examined by experienced gynecologists or dermatologists at the time of the visit to determine the presence of genital warts lesions in the cervix, vagina, vulva, anus, and perianal region of women, and genital warts lesions in the external genitalia, anus, and perianal region of men. If genital warts lesions were found, a sample preservation tube was used to collect biopsy tissue from the genital warts lesion and stored at 4°C until nucleic acid extraction.

### 2.3 Reagents and instruments

The viral genome extraction kit and human papilloma typing kit (HybriMax) were purchased from Guangdong Kaipu Biotechnology Co., Ltd. 21 HPV genotypes could be detected by the human papilloma typing kit (HybriMax), including 6 low-risk types (HPV-6, HPV-11, HPV-42, HPV-43, HPV-44, HPV-81) and 15 high-risk types (HPV-16, HPV-18, HPV-31, HPV-33, HPV-35, HPV-39, HPV-45, HPV-51, HPV-52, HPV-53, HPV-56, HPV-58, HPV-59, HPV-66, HPV-68). The main instruments included: TC-96/G/H(b) gene amplifier, which was produced by Hangzhou BORI Technology Co., Ltd; medical nucleic acid molecular rapid hybridizer HB-2012A and automatic nucleic acid extractor HBNP-4801A, which were produced by Guangdong Kaipu Biotechnology Co., Ltd.

### 2.4 DNA extraction, amplification and hybridization

(1) DNA was extracted from the samples by an automated nucleic acid extractor using a nucleic acid extraction kit (Magnetic Bead Method DR-4801-KZ). The quality of the extracted DNA was confirmed by amplifying the β-bead protein gene as an internal control. (2) 24 μL of prepared amplification reagent was added to the set PCR reaction tubes separately. 1 μL each of treated sample DNA, blank control, and positive control were added, respectively. The PCR amplification protocol was executed as follows: an initial hold at 20°C for 10 min, followed by a primary denaturation at 95°C for 9 min. This was succeeded by 40 cycles, each consisting of denaturation at 95°C for 20 s, annealing at 55°C for 30 s, and extension at 72°C for 30 s. Upon completion of these cycles, a final extension was conducted at 72°C for 5 min, with the reaction subsequently maintained at 4°C. (3) After completion of the amplification of the L region of the HPV genome, the membrane strips were labeled with the patient’s number and then fixed into the hybridizer, and hybridization and color development were carried out in accordance with the instruction manual. (4) Judgment of results: naked eye observation, the positive points and quality control points of the test results were clearly visible blue purple biotin staining points.

### 2.5 Principles of HPV genotype classification

The classification and statistics of HPV in this study were based on the following principles: (1) Based on the relationship between HPV and carcinogenicity: high-risk infection, low-risk infection, and co-infection (Infection caused by both HR-HPV and LR-HPV). (2) Based on the number of HPV genotypes infected: single infection and multiple infection. (3) Based on the HPV genotypes of licensed HPV vaccines: bivalent genotypes (2v) (HPV-16/18), quadrivalent genotypes (4v) (HPV-6/11/16/18), nine-valent genotypes (9v) (HPV-6/11/16/18/31/33/45/52/58), and non-9v genotypes (Non-9v) (HPV-42/43/44/81/35/39/51/53/56/59/66/68).

### 2.6 Statistical analysis

Participants grouping principles: (1) Participants were categorized into five groups according to their age (≤ 24, 25–34, 35–44, 45–54, and ≥ 55). (2) Participants were categorized into 8 groups based on the year of their visitation (2015, 2016, 2017, 2018, 2019, 2020, 2021, 2022). (3) Participants were categorized into 2 groups based on their gender (male and female). This study was conducted using WPS Office (2023) for data processing and analysis, and SPSS 25.0 statistical software was used to count the data. Count data were expressed as percentages (%) using the chi-square test. *P* < 0.05 was considered to indicate a statistically significant difference. The Goodman Kruskal Gamma method was used to analyze the correlation between HPV detection rate and age/year (ordered categorical variables), and calculated the Gamma coefficient. The Gamma coefficient ranges from −1 to 1, where *G* = 0 indicated two variables were uncorrelated, *G* > 0 indicated two variables were positively correlated, and *G* < 0 indicated two variables were negatively correlated. Chord plot, heat maps and bidirectional bar chart were plotted using the bioladder tools. Chord plot was used to display paired combinations of different types of HPV in patients with multiple infection. Heatmaps were used to visually display the differences in HPV genotype detection rates among different groups. Before drawing a heatmap, the data such as age and year were converted into detection rates. The data file was saved and imported into the heatmap tool of Bioloader, and the grouping information was imported into the *Y*-axis. Logarithmic processing, row clustering, and column clustering were not performed. After submission, a heatmap containing Z-values could be obtained. Using the Z-score method to normalize data with large differences by row could make the heatmap more vividly reflect the detection rate differences between different age and year groups of the same HPV type. Bidirectional bar chart was used to display the differences in HPV detection rates among different gender groups.

## 3 Results

### 3.1 HPV detection rate

Of the 1,625 patients with genital warts, 1,306 were detected as HPV positive, with a detection rate of 80.37% (1,306/1,625). The detection rates of HPV for low-risk infection, co-infection and high-risk infection were 49.48% (804/1,625), 24.92% (405/1,625) and 5.97% (97/1,625), respectively.

### 3.2 Single infection and multiple infection

**FIGURE 1:**
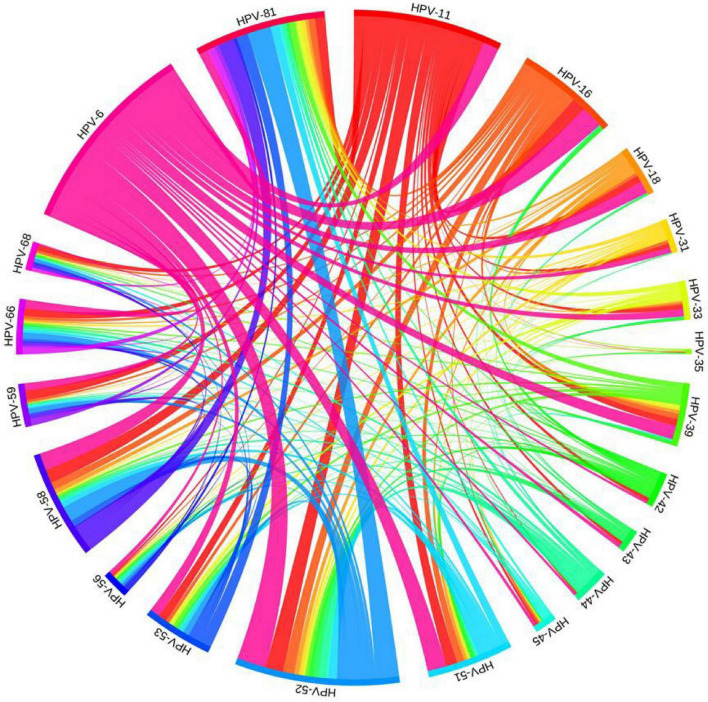
Chord plot of pairwise combinations of different HPV types in patients with multiple infection of genital warts in Foshan, China.

Of the 1,306 HPV infections, 844 (51.94%, 844/1,625) were caused by infection with a single HPV type, especially HPV-6 (23.75%, 386/1,625) and HPV-11 (14.46%, 235/1,625). Of the 462 (28.43%, 462/1,625) multiple infections, 280 (17.23%, 280/1,625) were double infections, 118 (7.26%, 118/1,625) were triple infections, 41 (2.52%, 41/1,625) were quadruple infections, and 23 (1.42%, 23/1,625) were quintuple or more infections. The detection rate of single infection was significantly higher than that of multiple infection (*P* < 0.05). Pairwise combinations of different HPV types in multiple infection could be clearly observed by chord plot (Figure 1), the most common combinations included HPV-6 and HPV-52 (47 times); HPV-52 and HPV-81 (38 times), and HPV-6 and HPV-16 (36 times).

### 3.3 HPV infection in patients with genital warts in different age groups

**FIGURE 2:**
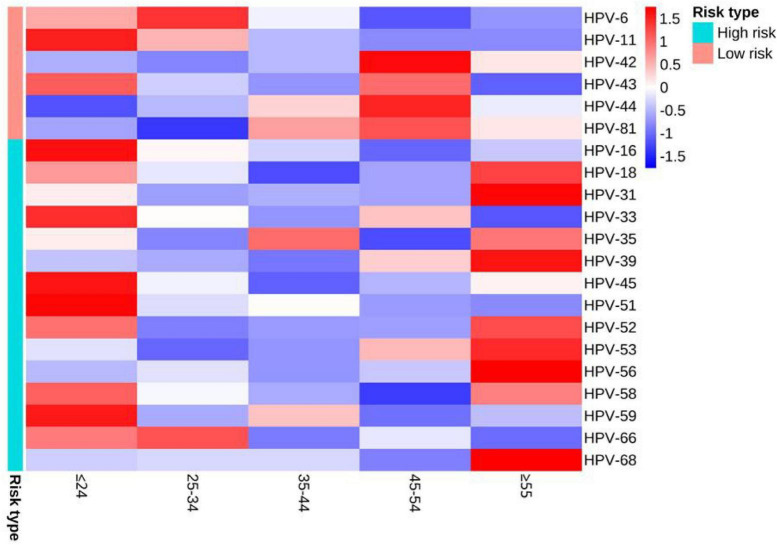
Heat map of the distribution of detection rates of HPV genotypes in different age groups. When the Z-value in the legend is lower than the average value, Z is negative, otherwise it is positive.

The detection rates of any HPV within the age groups ≤ 24, 25–34, 35–44, 45–54, and ≥ 55 were 82.11% (303/369), 79.69% (506/635), 81.33% (270/332), 82.86% (145/175) and 71.93% (82/114), respectively (Table 1). There were no statistically significant differences in the detection rates of any HPV, low-risk infection, co-infection, single infection and multiple infection among different age groups (all *P* > 0.05). The differences in detection rates of 3 types of LR-HPV (HPV-11, HPV-44, HPV-81) and 4 types of HR-HPV (HPV-16, HPV-33, HPV-39, HPV-51) were statistically significant (all *P* < 0.05). The detection rates of HPV-44 and HPV-81 tended to increase with ages (both *P* < 0.05), and the detection rates of high-risk infection, HPV-11, HPV-16, HPV-33, HPV-51, and HPV-58 trended to decrease with ages (all *P* < 0.05). The distribution of detection rates of HPV genotypes in different age groups can be seen by heat map (Figure 2).

**TABLE 1:** Detection rates of HPV in patients with genital warts in different age groups.

HPV genotype	Positive cases	≤ 24 (*n* = 369)	25–34 (*n* = 635)	35–44 (*n* = 332)	45–54 (*n* = 175)	≥ 55 (*n* = 114)	*P* ^a^	Gamma value	Gamma *P*^b^	Trend
Any HPV	1,306	303	506	270	145	82	0.205	−0.045	0.336	
High-risk infection	97	31	45	13	4	4	0.001	−0.284	< 0.001	Decreasing
Low-risk infection	804	155	333	176	98	42	0.416	0.062	0.093	
Co-infection	405	117	128	81	43	36	0.794	−0.039	0.386	
Single infection	844	171	356	180	97	40	0.459	0.007	0.861	
Multiple infection	462	132	150	90	48	42	0.767	−0.043	0.318	
HPV-6	572	131	241	111	53	36	0.1	−0.06	0.118	
HPV-11	366	107	150	61	29	19	< 0.001	−0.191	< 0.001	Decreasing
HPV-42	61	13	20	12	11	5	0.197	0.114	0.261	
HPV-43	31	11	10	4	5	1	0.307	−0.158	0.278	
HPV-44	66	6	22	18	15	5	0.001	0.327	< 0.001	Increasing
HPV-81	206	42	46	62	38	18	< 0.001	0.235	< 0.001	Increasing
HPV-16	92	33	34	15	5	5	0.005	−0.248	0.003	Decreasing
HPV-18	39	13	15	3	3	5	0.507	−0.147	0.273	
HPV-31	24	7	7	4	2	4	0.553	0.027	0.875	
HPV-33	31	14	11	2	4	0	0.01	−0.387	0.009	Decreasing
HPV-35	7	2	1	3	0	1	0.699	0.101	0.736	
HPV-39	51	11	16	5	9	10	0.008	0.19	0.11	
HPV-45	14	6	5	1	1	1	0.189	−0.322	0.152	
HPV-51	74	28	24	14	5	3	0.01	−0.242	0.01	Decreasing
HPV-52	148	46	47	26	14	15	0.619	−0.068	0.321	
HPV-53	51	13	14	9	8	7	0.125	0.112	0.336	
HPV-56	20	4	8	3	2	3	0.443	0.087	0.623	
HPV-58	93	31	35	14	4	9	0.071	−0.184	0.03	Decreasing
HPV-59	38	15	10	9	2	2	0.107	−0.209	0.112	
HPV-66	43	11	20	6	4	2	0.264	−0.136	0.235	
HPV-68	22	4	8	4	0	6	0.089	0.182	0.299	
2v	126	46	48	18	6	8	0.001	−0.272	< 0.001	Decreasing
4v	960	243	401	176	84	56	< 0.001	−0.196	< 0.001	Decreasing
9v	1,083	274	437	201	101	70	< 0.001	−0.186	< 0.001	Decreasing
Non-9v	223	29	69	69	44	12	< 0.001	0.283	< 0.001	Increasing

^a^The *P*-value is for difference in the detection rate of HPV among different age groups.

^b^The gamma *P*-value is for the correlation between the detection rate of HPV and different age groups.

### 3.4 HPV infection in patients with genital warts from 2015 to 2022

**FIGURE 3:**
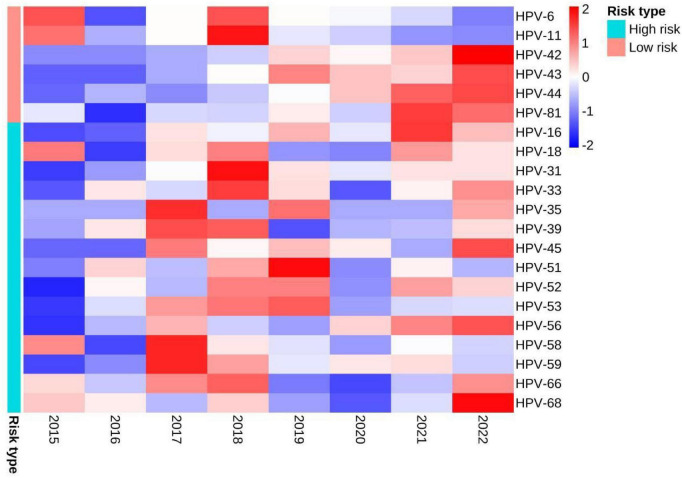
Heat map of the distribution of detection rates of HPV genotypes in different year groups. When the Z-value in the legend is lower than the average value, Z is negative, otherwise it is positive.

The detection rates of any HPV in 2015, 2016, 2017, 2018, 2019, 2020, 2011, and 2022 were 86.29% (107/124), 60.69% (88/145), 76.76% (109/142), 93.65% (118/126), 83.15% (153/184), 80.09% (189/236), 81.58% (248/304) and 80.77% (294/364), respectively (Table 2). There were no statistically significant differences in the detection rates of any HPV, high-risk infection, low-risk infection and single infection among different year groups (all *P* > 0.05). However, the differences in detection rates of co-infection and multiple infection were statistically significant (both *P* < 0.05). The differences in detection rates of 4 types of LR-HPV (HPV-11, HPV-42, HPV-43, HPV-44) and 1 type of HR-HPV (HPV-16) were statistically significant (all *P* < 0.05). The detection rates of co-infection, multiple infection, HPV-42, HPV-43, HPV-44 and HPV-16 tended to increase with years (all *P* < 0.05), and the detection rates of HPV-6 and HPV-11 trended to decrease with years (both *P* < 0.05). The distribution of detection rates of HPV genotypes in different year groups can be seen by heat map (Figure 3).

**TABLE 2:** Detection rates of HPV in patients with genital warts from 2015 to 2022.

HPV genotype	Positive cases	2015 (*n* = 124)	2016 (*n* = 145)	2017 (*n* = 142)	2018 (*n* = 126)	2019 (*n* = 184)	2020 (*n* = 236)	2021 (*n* = 304)	2022 (*n* = 364)	*P* ^a^	Gamma value	Gamma *P*^b^	Trend
Any HPV	1,306	107	88	109	118	153	189	248	294	0.097	0.055	0.195	
High-risk infection	97	7	5	12	10	13	9	21	20	0.945	−0.011	0.872	
Low-risk infection	804	81	57	61	68	91	135	141	170	0.256	−0.042	0.203	
Co-infection	405	19	26	36	40	49	45	86	104	0.004	0.107	0.005	Increasing
Single infection	844	85	58	67	69	96	138	152	179	0.307	−0.037	0.258	
Multiple infection	462	22	30	42	49	57	51	96	115	0.01	0.089	0.014	Increasing
HPV-6	572	55	41	52	56	67	85	105	111	0.055	−0.074	0.032	Decreasing
HPV-11	366	36	30	34	41	42	52	60	71	0.017	−0.097	0.014	Decreasing
HPV-42	61	0	0	1	2	7	7	12	32	< 0.001	0.551	< 0.001	Increasing
HPV-43	31	0	0	1	2	5	5	6	12	0.003	0.362	0.002	Increasing
HPV-44	66	1	3	2	3	6	10	18	23	< 0.001	0.338	< 0.001	Increasing
HPV-81	206	15	15	17	15	23	28	43	50	0.26	0.058	0.247	
HPV-16	92	2	3	8	6	12	11	27	23	0.004	0.193	0.004	Increasing
HPV-18	39	5	0	4	5	2	2	11	10	0.634	0.073	0.524	
HPV-31	24	0	1	2	4	3	3	5	6	0.313	0.115	0.357	
HPV-33	31	0	3	2	5	4	0	6	11	0.215	0.168	0.188	
HPV-35	7	0	0	2	0	2	0	0	3	0.686	0.121	0.657	
HPV-39	51	3	5	7	6	3	6	8	13	0.771	−0.014	0.884	
HPV-45	14	0	0	2	1	2	2	1	6	0.163	0.263	0.157	
HPV-51	74	3	8	5	8	17	6	15	12	0.636	−0.065	0.374	
HPV-52	148	4	13	10	15	22	14	34	36	0.084	0.089	0.108	
HPV-53	51	0	4	7	7	11	4	8	10	0.917	−0.04	0.642	
HPV-56	20	0	1	2	1	1	3	5	7	0.066	0.292	0.056	
HPV-58	93	10	4	14	8	10	10	18	19	0.374	−0.058	0.421	
HPV-59	38	1	2	6	4	4	6	8	7	0.825	0.002	0.988	
HPV-66	43	4	3	6	6	2	1	6	15	0.96	0.041	0.729	
HPV-68	22	2	2	1	2	1	0	3	11	0.224	0.256	0.152	
2v	126	7	3	9	10	14	12	38	33	0.002	0.19	0.002	Increasing
4v	960	91	71	84	98	115	140	174	187	0.001	−0.12	< 0.001	Decreasing
9v	1,083	94	78	99	109	126	156	200	221	0.03	−0.09	0.011	Decreasing
Non-9v	223	13	10	10	9	27	33	48	73	< 0.001	0.247	< 0.001	Increasing

^a^The *P*-value is for difference in the detection rate of HPV among different year groups.

^b^The gamma *P*-value is for the correlation between the detection rate of HPV and different year groups.

### 3.5 HPV infection in patients with genital warts in different gender groups

**FIGURE 4:**
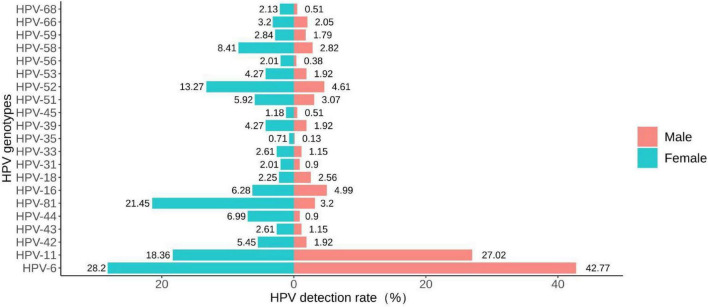
Bidirectional bar chart of the distribution of detection rates of HPV genotypes in different gender groups.

The detection rates of any HPV in the male and the female groups were 76.70% (599/781) and 83.77% (707/844), respectively (Table 3). There were no statistically significant differences in the detection rates of high-risk infection, HPV-16, HPV-18, HPV-31, HPV-35, HPV-45, HPV-59 and HPV-66 between the two groups (all *P* > 0.05). The detection rates of low-risk infection, single infection, HPV-6 and HPV-11 in the male group were higher than that in the female group (all *P* < 0.05). The detection rates of any HPV, co-infection, multiple infection, HPV-42, HPV-43, HPV-44, HPV-81, HPV-33, HPV-39, HPV-51, HPV-52, HPV-53, HPV-56, HPV-58 and HPV-68 in the female group were higher than that in the male group (all *P* < 0.05). The distribution of the detection rates of HPV genotypes in different gender groups can be seen by the bidirectional bar chart (Figure 4).

**TABLE 3:** Detection rates of HPV in patients with genital warts in different gender groups.

HPV genotype	Positive cases	Male (*n* = 781)	Female (*n* = 844)	All detection rate (%)	Male detection rate (%)	Female detection rate (%)	χ^2^	*P*
Any HPV	1,306	599	707	80.37%	76.70%	83.77%	12.856	< 0.001
High-risk infection	97	40	57	5.97%	5.12%	6.75%	1.925	0.165
Low-risk infection	804	437	367	49.48%	55.95%	43.48%	25.236	< 0.001
Co-infection	405	122	283	24.92%	15.62%	33.53%	69.537	< 0.001
Single infection	844	451	393	51.94%	57.75%	46.56%	20.32	< 0.001
Multiple infection	462	148	314	28.43%	18.95%	37.20%	66.424	< 0.001
HPV-6	572	334	238	35.20%	42.77%	28.20%	37.735	< 0.001
HPV-11	366	211	155	22.52%	27.02%	18.36%	17.4	< 0.001
HPV-42	61	15	46	3.75%	1.92%	5.45%	13.987	< 0.001
HPV-43	31	9	22	1.91%	1.15%	2.61%	4.584	0.032
HPV-44	66	7	59	4.06%	0.90%	6.99%	38.663	< 0.001
HPV-81	206	25	181	12.68%	3.20%	21.45%	121.972	< 0.001
HPV-16	92	39	53	5.66%	4.99%	6.28%	1.256	0.262
HPV-18	39	20	19	2.40%	2.56%	2.25%	0.166	0.684
HPV-31	24	7	17	1.48%	0.90%	2.01%	3.484	0.062
HPV-33	31	9	22	1.91%	1.15%	2.61%	4.584	0.032
HPV-35	7	1	6	0.43%	0.13%	0.71%	3.213	0.073
HPV-39	51	15	36	3.14%	1.92%	4.27%	7.336	0.007
HPV-45	14	4	10	0.86%	0.51%	1.18%	2.149	0.143
HPV-51	74	24	50	4.55%	3.07%	5.92%	7.587	0.006
HPV-52	148	36	112	9.11%	4.61%	13.27%	36.754	< 0.001
HPV-53	51	15	36	3.14%	1.92%	4.27%	7.336	0.007
HPV-56	20	3	17	1.23%	0.38%	2.01%	8.867	0.003
HPV-58	93	22	71	5.72%	2.82%	8.41%	23.538	< 0.001
HPV-59	38	14	24	2.34%	1.79%	2.84%	1.962	0.161
HPV-66	43	16	27	2.65%	2.05%	3.20%	2.084	0.149
HPV-68	22	4	18	1.35%	0.51%	2.13%	7.976	0.005
2v	126	55	71	7.75%	7.04%	8.41%	1.065	0.302
4v	960	551	409	59.08%	70.55%	48.46%	81.88	< 0.001
9v	1,083	568	515	66.65%	72.73%	61.02%	25.015	< 0.001
Non-9v	223	31	192	13.72%	3.97%	22.75%	120.827	< 0.001

### 3.6 Presence of licensed HPV vaccine genotypes

**FIGURE 5:**
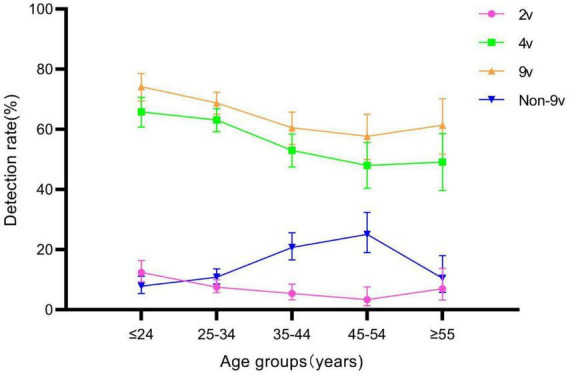
Genotype detection rates of 2v, 4v, 9v, and Non-9v among different age groups. Error bars represent 95% confidence intervals.

The detection rates of 2v, 4v, 9v, and Non-9v genotypes were 7.75% (126/1,625), 59.08% (960/1,625), 66.65% (1,083/1,625), and 13.72% (223/1,625), respectively (Table 3). There were statistically significant differences in genotype detection rates of 2v, 4v, 9v, and Non-9v among different age groups (all *P* < 0.05) (Table 1). The genotype detection rates of 2v, 4v and 9v showed a decreasing trend with ages (all *P* < 0.05), while the genotype detection rate of Non-9v showed an increasing trend with ages (*P* < 0.05) (Figure 5).

**FIGURE 6:**
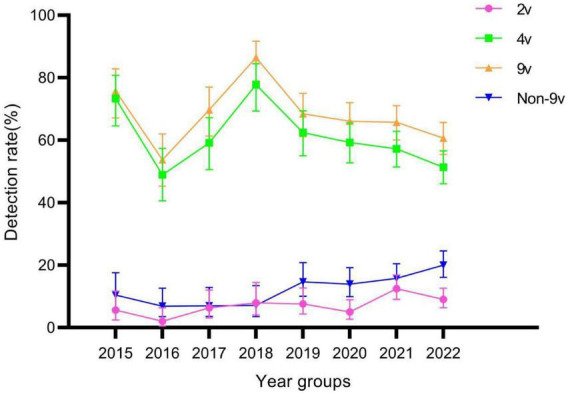
Genotype detection rates of 2v, 4v, 9v, and Non-9v among different year groups. Error bars represent 95% confidence intervals.

There were statistically significant differences in genotype detection rates of 2v, 4v, 9v, and Non-9v among different year groups (all *P* < 0.05) (Table 2). The genotype detection rates of 2v and Non-9v showed an increasing trend from 2015 to 2022 (both *P* < 0.05), while the genotype detection rates of 4v and 9v showed a decreasing trend from 2015 to 2022 (both *P* < 0.05) (Figure 6).

We compared the differences in genotype detection rates of 2v, 4v, 9v and Non-9v between genders. The genotype detection rates of 4v and 9v in the male group were higher than those in the female group (both *P* < 0.05). The genotype detection rate of Non-9v in the female group was higher than that in the male group (*P* < 0.05) (Table 3). We also compared the differences in genotype detection rates among 2v, 4v and 9v of the same gender (Table 4). The genotype detection rates of 4v and 9v in the male group were significantly higher than that of 2v (both *P* < 0.05). However, there was no statistically significant difference in the genotype detection rate between 4v and 9v (*P* > 0.05) in the male group. The genotype detection rates among 2v, 4v, and 9v in the female group showed statistically significant differences (all *P* < 0.05).

**TABLE 4:** Genotype detection rates among 2v, 4v and 9v in patients with genital warts of the same gender.

	2v	4v	9v	*P*		
				**2v and 4v**	**2v and 9v**	**4v and 9v**
Male	55 (7.04%)	551 (70.55%)	568 (72.73%)	< 0.001	< 0.001	0.34
Female	71 (8.41%)	409 (48.46%)	515 (61.02%)	< 0.001	< 0.001	< 0.001

## 4 Discussion

Comparative studies of the HPV distribution in patients with genital warts of both sexes in the Asia-Pacific region are limited, with even fewer reports from China. At present, researches on the distribution of HPV in China mainly focus on the female population (Chen et al., 2023; Wei et al., 2023; Zhang et al., 2023); Research on the distribution of HPV among general warts is commonly found in northern China (Zhu C. et al., 2019). HPV infection is mainly transmitted between male and female, and the distribution of HPV also has regional characteristics. On the other hand, based on the HPV infection status in patients with genital warts in both genders, research on the genotype detection rate of licensed HPV vaccines is also very rare. This long-term study of 1,625 patients with genital warts in southern China, provided information about HPV infection in Chinese patients with genital warts by age groups, year groups, gender groups, and different genotypes of licensed HPV vaccine groups. The aim was to evaluate the prevalence of human papillomavirus (HPV) infection and presence of licensed HPV vaccine genotypes among genital wart patients. We acknowledge that a high HPV DNA load may indeed reflect elevated viral DNA replication activity, which could correlate with more severe infection states. However, we also highlight the inherent challenges associated with quantifying HPV DNA load due to the heterogenous nature of sampling. Unlike plasma, genital wart samples present difficulties in achieving precise quantification, which influenced our decision to employ qualitative PCR for sample detection. Our study employed infusion hybridization for HPV typing, a method known for its high sensitivity and specificity. This choice was based on the method’s established reliability for detecting a wide range of HPV genotypes. Given the large sample size and the long-term periodic analysis conducted, we opted not to use DNA sequencing for additional genotype verification, considering the strengths of the chosen method. While our study based on hybridization methods has provided valuable information on the epidemiological characteristics of HPV, we acknowledge that more effective methods such as next-generation sequencing (NGS) could offer more detailed HPV genotype information, including co-infections and minor genetic variations, enabling us to more accurately describe the associations between HPV genotypes and various factors. Moreover, to prevent nucleic acid contamination that could affect the outcomes of multiple infections, we handled samples under stringent anti-contamination conditions, used quality control samples, repeated all dubious results, and conducted regular environmental cleaning to ensure the consistency and accuracy of our results.

The detection rate of any HPV was 80.37%. To clarify, the overall undetected rate of HPV in our study was 19.63%. This undetection may be attributed to several factors including the nature of the sample type (e.g., samples from the cervix, vagina, vulva, anus, and perianal region in women, and from lesions in the external genitalia, anus, and perianal region in men), the methodology employed for HPV detection, and the limitations inherent to the sampling process itself. Additionally, the range of HPV types that can be detected by our methodology may also contribute to this outcome. It’s noteworthy that similar studies in the past have reported undetection rates within this range (Ingles et al., 2015; Yuan et al., 2023), suggesting that our findings are consistent with the broader body of research. To mitigate the undetected rate, future research should consider employing other more sensitive and extensive methods to verify HPV negative results. As seen from the chord plot, 21 HPV genotypes were detected in multiple infection. The number of samples with high-risk HPV was quite high in patients with genital warts, which is consistent with the results of a previous study (Zare-Bidaki et al., 2022). Some studies have reported that high-risk HPV genotypes may also be associated with warts (Garland et al., 2009; Hasanzadeh et al., 2019). In this study, co-infections and multiple infections were analyzed separately because, according to the HPV genotype classification criteria in this paper, co-infection is caused by a combination of high-risk and low-risk infections. The presence of high-risk types brings a risk of carcinogenesis. Multiple infections, on the other hand, focus on the number of HPV genotypes, without distinguishing between low-risk and high-risk types.

### 4.1 Age specificity of HPV infection

Our study found that 82.22% (1,336/1,625) of the cases had an age distribution of ≤ 24, 25–34, and 35–44, which may be related to the level of sexual activity. This study found no significant difference in HPV detection rates among different age groups, which is different from a previous study of high-risk male HPV infected individuals (Yin et al., 2020), This may be related to the fact that the research subjects of this study are male and female patients with genital warts. In addition, long-term and persistent HPV infection may pose a risk of cancer transformation, so it is necessary to conduct comprehensive and regular screening for elderly people infected with HPV (Sudenga et al., 2017). Our study also showed that the detection rates of some HPV genotypes were age-specific. Due to cost and technical difficulties, licensed HPV vaccines cannot cover all HPV genotypes. But if different HPV vaccines are designed for different age groups, it may be possible to solve the above problem.

### 4.2 Annual specificity of HPV infection

Current literature studies on annual trends in HPV have focused on HPV infection in women (Li et al., 2022), and there are fewer studies on annual trends in patients with genital warts. Our study found no significant difference in the detection rate of any HPV among patients with genital warts from 2015 to 2022. However, the detection rates of co-infection, multiple infection, HPV-42, HPV-43, HPV-44, and HPV-16 showed a gradual increased over time. This differs from another study on the annual distribution of HPV genotypes in male genital warts patients in Shanghai (Li et al., 2021). This suggests that there might be other factors influencing the annual detection rates of HPV infections, including but not limited to trends in the prevalence of each HPV subtype, the impact of HPV vaccination, patients’ sexual behavior habits, and other environmental or societal factors not directly considered in our study. It is worth noting that the currently approved HPV vaccines do not include the low-risk HPV-81 as well as HPV-42, HPV-43, and HPV-44 with increasing detection rates year by year.

### 4.3 Gender specificity of HPV infection

Our study found that the detection rate of any HPV was higher in the female group than in the male group. A similar study from Xi’an found that the detection rate of any HPV was higher in males than in females. However, the detection rate of LR-HPV was significantly higher in men than in women (52.3 vs. 35.7%, *p* < 0.01), which was consistent with this study (Zhu C. et al., 2019). A study suggested that the incidence or clearance of HPV infection may be higher in females than in males due to differences in the biological structure between males and females (Widdice et al., 2010). Our study found that the most common genotypes for males are HPV-6 (334/781, 42.77%) and HPV-11 (211/781, 27.02%), while the most common genotypes for females are HPV-6 (238/844, 28.20%) and HPV-81 (181/844, 21.45%). In contrast, research results from Shandong Province showed that the common genotypes for both males and females in patients with genital warts were HPV-6 and HPV-11 (Yuan et al., 2023).

### 4.4 Licensed HPV vaccine genotypes

The human papillomavirus vaccine was originally developed to reduce the number of HPV-related cancers and precancerous lesions in women. Related studies have also confirmed that the incidence of genital warts in young heterosexuals could be reduced by HPV vaccination (Read et al., 2011; Chow et al., 2015). Based on the three main vaccine types currently produced globally (2v, 4v, and 9v) (Luckett and Feldman, 2016), we statistically analyzed the HPV detection rates of their HPV genotypes. The genotype detection rates of 2v, 4v and 9v showed a decreasing trend with ages. From 2015 to 2022, the genotypes detection rates of 4v and 9v in patients with genital warts showed a decreasing trend. However, the genotype detection rate of 2v tended to increase. This may be due to the fact that 2v vaccine does not include HPV-6 and HPV-11, which cause genital warts. Therefore, we can hypothesize that as vaccination with the 4v and 9v vaccines becomes more prevalent over time, leading to a decreasing detection rate of HPV-6 and HPV-11 in the population with genital warts. On the other hand, the increase in HPV-16 detection rate in 2v vaccine is not mainly due to genital warts, but may coexist with low-risk HPV types. We also found that the genotype detection rates of 4v and 9v in the male group were higher than those in the female group, which was opposite to the genotype detection rate of any HPV. The reason was that the genotype detection rate of Non-9v in the female group was higher than that in the male group. The higher detection rate of Non-9v genotypes in females may reflect the protective effect of vaccination against specific HPV types, though it’s unclear if all females tested had received the vaccine. Future research could further collect patient vaccination information, allowing for a more accurate interpretation of these findings. It indicated that females with genital warts were more likely to be infected with more types of HPV. In addition, the genotype detection rate of 9v was significantly higher than that of 2v and 4v in the female group. However, as in the United States (Hirth, 2019), the existing HPV vaccination policy only allows girls aged 13–15 years to receive free vaccinations in specific areas in China (Zhang et al., 2022). The insufficient supply of HPV 9v vaccine, coupled with the high cost of the vaccine (Chen et al., 2021), have led to difficulties in promoting the HPV vaccine in China. Similar to previous studies (Schlecht et al., 2021; Yuan et al., 2023), HPV-42, HPV-44, and HPV-81, as the high prevalence genotypes in this study, have not been included in any licensed HPV vaccines. Therefore, it is necessary to develop a wider range of HPV vaccine (Castle and Maza, 2016). In developed countries that adopt male HPV vaccination, cost-effectiveness models indicate the superiority of incorporating male vaccination (Lin et al., 2017).

## 5 Conclusion

In conclusion, our study reveals that low-risk HPV infections and single HPV infections predominate among patients with genital warts, highlighting the significant burden of these infections within the age group of 15 to 44 years. The prevention and diagnosis of genital warts should fully consider factors such as age, year and gender. These findings underscore the urgent need for the development and implementation of broader-spectrum HPV vaccines. Additionally, our data suggest the importance of expanding HPV vaccination programs to include males, as a strategy to reduce the incidence of genital warts. This approach not only targets the prevention of HPV-related diseases in women but also addresses the transmission dynamics of HPV infections, thereby contributing to the overall reduction of HPV prevalence in the population.

## Data availability statement

The raw data supporting the conclusions of this article will be made available by the authors, without undue reservation.

## Ethics statement

The studies involving humans were approved by the Ethics Committee of the Second People’s Hospital of Foshan. The studies were conducted in accordance with the local legislation and institutional requirements. Written informed consent for participation in this study was provided by the participants’ legal guardians/next of kin. Written informed consent was obtained from the individual(s), and minor(s)’ legal guardian/next of kin, for the publication of any potentially identifiable images or data included in this article.

## Author contributions

ZH: Conceptualization, Data curation, Visualization, Writing – original draft, Writing – review & editing. SY: Conceptualization, Data curation, Writing – original draft, Writing – review & editing. LZ: Formal analysis, Writing – original draft, Writing – review & editing. WX: Formal analysis, Writing – original draft, Writing – review & editing. DX: Formal analysis, Writing – original draft, Writing – review & editing. WL: Formal analysis, Writing – original draft, Writing – review & editing. DT: Data curation, Writing – original draft, Writing – review & editing. JS: Data curation, Writing – original draft, Writing – review & editing.

## References

[B1] Bhagavathula A. S. Massey P. M. (2022). Google trends on human papillomavirus vaccine searches in the United States From 2010 to 2021: Infodemiology study. *JMIR Public Health Surveill.* 8:e37656. 10.2196/37656 36036972 PMC9468915

[B2] Bruni L. Albero G. Rowley J. Alemany L. Arbyn M. Giuliano A. R. (2023). Global and regional estimates of genital human papillomavirus prevalence among men: A systematic review and meta-analysis. *Lancet Glob. Health* 11 e1345–e1362. 10.1016/S2214-109X(23)00305-4 37591583 PMC10447222

[B3] Castle P. E. Maza M. (2016). Prophylactic HPV vaccination: Past, present, and future. *Epidemiol. Infect.* 144 449–468. 10.1017/S0950268815002198 26429676

[B4] Chen G. Wu B. Dai X. Zhang M. Liu Y. Huang H. (2021). Gender differences in knowledge and attitude towards HPV and HPV vaccine among college students in Wenzhou, China. *Vaccines* 10:10. 10.3390/vaccines10010010 35062671 PMC8779512

[B5] Chen Q. Qu W. Zhao Y. Shu L. Wang Y. Chen X. (2023). The prevalence of HPV among 164,137 women in China exhibited some unique epidemiological characteristics. *Infect. Agents Cancer* 18:72. 10.1186/s13027-023-00553-4 37950328 PMC10638728

[B6] Chow E. P. Read T. R. Wigan R. Donovan B. Chen M. Y. Bradshaw C. S. (2015). Ongoing decline in genital warts among young heterosexuals 7 years after the Australian human papillomavirus (HPV) vaccination programme. *Sex. Transm. Infect.* 91 214–219. 10.1136/sextrans-2014-051813 25305210

[B7] Crow J. M. (2012). HPV: The global burden. *Nature* 488 S2–S3. 10.1038/488S2a 22932437

[B8] Garland S. M. Steben M. Sings H. L. James M. Lu S. Railkar R. (2009). Natural history of genital warts: Analysis of the placebo arm of 2 randomized phase III trials of a quadrivalent human papillomavirus (types 6, 11, 16, and 18) vaccine. *J. Infect. Dis.* 199 805–814. 10.1086/597071 19199546

[B9] Geretti A. M. Brook G. Cameron C. Chadwick D. French N. Heyderman R. (2016). British HIV association guidelines on the use of vaccines in HIV-positive adults 2015. *HIV Med.* 17 (Suppl. 3), s2–s81. 10.1111/hiv.12424 27568789

[B10] Hasanzadeh M. Rejali M. Mehramiz M. Akbari M. Mousavi Seresht L. Yazdandoost Y. (2019). The interaction of high and low-risk human papillomavirus genotypes increases the risk of developing genital warts: A population-based cohort study. *J. Cell. Biochem.* 120 12870–12874. 10.1002/jcb.28557 30868650

[B11] Hirth J. (2019). Disparities in HPV vaccination rates and HPV prevalence in the United States: A review of the literature. *Hum. Vaccin. Immunother.* 15 146–155. 10.1080/21645515.2018.1512453 30148974 PMC6363146

[B12] Ingles D. J. Pierce Campbell C. M. Messina J. A. Stoler M. H. Lin H. Y. Fulp W. J. (2015). Human papillomavirus virus (HPV) genotype- and age-specific analyses of external genital lesions among men in the HPV Infection in Men (HIM) Study. *J. Infect. Dis.* 211 1060–1067. 10.1093/infdis/jiu587 25344518 PMC4432433

[B13] Li M. Zhao C. Zhao Y. Li J. Wei L. (2023). Immunogenicity, efficacy, and safety of human papillomavirus vaccine: Data from China. *Front. Immunol.* 14:1112750. 10.3389/fimmu.2023.1112750 36993948 PMC10040563

[B14] Li X. Xiang F. Chen Z. Zhang T. Zhu Z. Zhang M. (2021). Genital Human Papillomavirus Prevalence and Genotyping Among Males in Putuo District of Shanghai, China 2015-2019. *MEDICAL SCIENCE MONITOR* 27 e932093. 10.12659/MSM.932093 34475371 PMC8422898

[B15] Li X. Xiang F. Dai J. Zhang T. Chen Z. Zhang M. (2022). Prevalence of cervicovaginal human papillomavirus infection and genotype distribution in Shanghai, China. *Virol. J.* 19:146. 10.1186/s12985-022-01879-y 36096810 PMC9465878

[B16] Lin A. Ong K. J. Hobbelen P. King E. Mesher D. Edmunds W. J. (2017). Impact and cost-effectiveness of selective human papillomavirus vaccination of men who have sex with men. *Clin. Infect. Dis.* 64 580–588. 10.1093/cid/ciw845 28011615 PMC5404831

[B17] Luckett R. Feldman S. (2016). Impact of 2-, 4- and 9-valent HPV vaccines on morbidity and mortality from cervical cancer. *Hum. Vaccin. Immunother.* 12 1332–1342. 10.1080/21645515.2015.1108500 26588179 PMC4964711

[B18] Malagutti N. Rotondo J. C. Cerritelli L. Melchiorri C. De Mattei M. Selvatici R. (2020). High human papillomavirus DNA loads in inflammatory middle ear diseases. *Pathogens* 9:224. 10.3390/pathogens9030224 32197385 PMC7157545

[B19] Medda A. Duca D. Chiocca S. (2021). Human papillomavirus and cellular pathways: Hits and targets. *Pathogens* 10:262. 10.3390/pathogens10030262 33668730 PMC7996217

[B20] Owczarek W. Slowinska M. Walecka I. Ciazynska M. Nowicka D. Walczak L. (2021). Correlation of the ALA-PDT treatment efficacy and the HPV genotype profile of genital warts after cryotherapy failure and podophyllotoxin therapy in male patients. *Life* 11:146. 10.3390/life11020146 33672889 PMC7918501

[B21] Read T. R. Hocking J. S. Chen M. Y. Donovan B. Bradshaw C. S. Fairley C. K. (2011). The near disappearance of genital warts in young women 4 years after commencing a national human papillomavirus (HPV) vaccination programme. *Sex. Transm. Infect.* 87 544–547. 10.1136/sextrans-2011-050234 21970896

[B22] Schlecht N. F. Diaz A. Nucci-Sack A. Shyhalla K. Shankar V. Guillot M. (2021). Incidence and types of human papillomavirus infections in adolescent girls and young women immunized with the human papillomavirus vaccine. *JAMA Netw. Open* 4:e2121893. 10.1001/jamanetworkopen.2021.21893 34424304 PMC8383132

[B23] Smith M. A. Liu B. McIntyre P. Menzies R. Dey A. Canfell K. (2015). Fall in genital warts diagnoses in the general and indigenous Australian population following implementation of a national human papillomavirus vaccination program: Analysis of routinely collected national hospital data. *J. Infect. Dis.* 211 91–99. 10.1093/infdis/jiu370 25117753

[B24] Stanley M. (2007). Prophylactic HPV vaccines: Prospects for eliminating ano-genital cancer. *Br. J. Cancer* 96 1320–1323. 10.1038/sj.bjc.6603695 17375045 PMC2360190

[B25] Steben M. Garland S. M. (2014). Genital warts. *Best Pract. Res. Clin. Obstet. Gynaecol.* 28 1063–1073. 10.1016/j.bpobgyn.2014.07.002 25155525

[B26] Sudenga S. L. Torres B. N. Silva R. Villa L. L. Lazcano-Ponce E. Abrahamsen M. (2017). Comparison of the natural history of genital HPV infection among men by country: Brazil, Mexico, and the United States. *Cancer Epidemiol. Biomark. Prev.* 26 1043–1052. 10.1158/1055-9965.EPI-17-0040 28446543 PMC5556383

[B27] Wei X. Zhang J. Mei Y. Dai Q. Yang X. Wang X. (2023). Prevalence and genotype distribution of HPV6/11/16/18 infections among 180,276 outpatient females from a Women’s and Children’s Central Hospital, 2015-2021, Chengdu, China. *Sci. Rep.* 13:22249. 10.1038/s41598-023-48222-1 38097632 PMC10721790

[B28] Widdice L. E. Breland D. J. Jonte J. Farhat S. Ma Y. Leonard A. C. (2010). Human papillomavirus concordance in heterosexual couples. *J. Adolesc. Health* 47 151–159. 10.1016/j.jadohealth.2010.01.006 20638007 PMC2967294

[B29] World Health Organization [WHO] (2017). Human papillomavirus vaccines: WHO position paper, May 2017-Recommendations. *Vaccine* 35 5753–5755. 10.1016/j.vaccine.2017.05.069 28596091

[B30] Yin W. G. Yang M. Peng L. Liu Y. M. Cheng B. Xuan S. X. (2020). Male papillomavirus infection and genotyping in the Qingyuan area. *Virol. J.* 17:155. 10.1186/s12985-020-01423-w 33076966 PMC7574239

[B31] Yuan H. Li R. Lv J. Yi G. Sun X. Zhao N. (2023). Epidemiology of human papillomavirus on condyloma acuminatum in Shandong Province, China. *Hum. Vaccin. Immunother.* 19:2170662. 10.1080/21645515.2023.2170662 36919446 PMC10064924

[B32] Zare-Bidaki M. Zardast M. Nadjafi-Semnani A. Nadjafi-Semnani M. Javanmard D. Ghafari S. (2022). Investigation of frequency and typing of human papillomavirus among genital warts using a reverse dot blot hybridization approach. *BMC Infect. Dis.* 22:278. 10.1186/s12879-022-07276-8 35317740 PMC8941769

[B33] Zhang W. Guo N. Li B. Shang E. Wang J. Zhang M. (2023). Prevalence and genotype distribution of human papillomavirus infections in Beijing, China between 2016 and 2020. *Virol. J.* 20:11. 10.1186/s12985-023-01959-7 36653807 PMC9847084

[B34] Zhang X. X. Wang W. Song Y. F. Zhang Z. N. Yu W. Z. (2022). [Expert recommendations on human papillomavirus vaccine immunization strategies in China]. *Zhonghua Yu Fang Yi Xue Za Zhi* 56 1165–1174. 10.3760/cma.j.cn112150-20220505-00443 36207876

[B35] Zhu B. Liu Y. Zuo T. Cui X. Li M. Zhang J. (2019). The prevalence, trends, and geographical distribution of human papillomavirus infection in China: The pooled analysis of 1.7 million women. *Cancer Med.* 8 5373–5385. 10.1002/cam4.2017 31350872 PMC6718589

[B36] Zhu C. Wang Y. Mao W. Zhang H. Ma J. (2019). Prevalence and distribution of HPV types in genital warts in Xi’an, China: A prospective study. *BMJ Open* 9:e023897. 10.1136/bmjopen-2018-023897 31092642 PMC6530368

[B37] Zou K. Huang Y. Li Z. (2022). Prevention and treatment of human papillomavirus in men benefits both men and women. *Front. Cell Infect. Microbiol.* 12:1077651. 10.3389/fcimb.2022.1077651 36506029 PMC9729793

